# Supplementary material for: miR-34/449 miRNAs regulate choroid plexus ciliogenesis to control cerebrospinal fluid production
Source: bioRxiv. 2026 Sep 1:2026.08.30.747939. Preprint. [Version 1] doi: 10.64898/2026.08.30.747939 (PMC13684506; doi:10.64898/2026.08.30.747939)
Supplement: Supplementary file 1 [file NIHPP2026.08.30.747939v1-supplement-1.pdf]

bioRxiv preprint doi: https://doi.org/10.64898/2026.08.30.747939; this version posted September 1, 2026. The copyright holder for this preprint (which was not certified by peer review) is the author/funder, who has granted bioRxiv a license to display the preprint in perpetuity. It is made available under a CC-BY 4.0 International license.

Supplementary Reference

1. Song, R., Walentek, P., Sponer, N., Klimke, A., Lee, J.S., Dixon, G., Harland, R., Wan, Y., Lishko, P., Lize, M., et al. (2014). miR-34/449 miRNAs are required for motile ciliogenesis by repressing cp110. Nature *510*, 115–120. https://doi.org/10.1038/nature13413.

2. Christensen, I.B., Gyldenholm, T., Damkier, H.H., and Praetorius, J. (2013). Polarization of membrane associated proteins in the choroid plexus epithelium from normal and slc4a10 knockout mice. Front. Physiol. *4*. https://doi.org/10.3389/fphys.2013.00344.

3. Monnot, A.D., and Zheng, W. (2012). Culture of Choroid Plexus Epithelial Cells and In Vitro Model of Blood–CSF Barrier. In Epithelial Cell Culture Protocols Methods in Molecular Biology., S. H. Randell and M. L. Fulcher, eds. (Humana Press), pp. 13–29. https://doi.org/10.1007/978-1-62703-125-7_2.

4. Hochstetler, A., Hulme, L., Delpire, E., Schwerk, C., Schroten, H., Preston, D., Simpson, S., and Blazer-Yost, B.L. (2022). Porcine choroid plexus-Riems cell line demonstrates altered polarization of transport proteins compared with the native epithelium. Am. J. Physiol.-Cell Physiol. *323*, C1–C13. https://doi.org/10.1152/ajpcell.00374.2021.

5. Cooney, A.L., Thurman, A.L., McCray, P.B., Pezzulo, A.A., and Sinn, P.L. (2021). Lentiviral vectors transduce lung stem cells without disrupting plasticity. Mol. Ther. - Nucleic Acids *25*, 293–301. https://doi.org/10.1016/j.omtn.2021.06.010.

6. Hao, S., Zhang, X., Ning, K., Feng, Z., Park, S.Y., Aksu Kuz, C., McFarlin, S., Richart, D., Cheng, F., Zhang, E.Y., et al. (2023). Identification of host essential factors for recombinant AAV transduction of the polarized human airway epithelium. J. Virol. *97*, e01330-23. https://doi.org/10.1128/jvi.01330-23.

7. Hamilton, B.A., Li, X., Pezzulo, A.A., Abou Alaiwa, M.H., and Zabner, J. (2019). Polarized AAVR expression determines infectivity by AAV gene therapy vectors. Gene Ther. *26*, 240–249. https://doi.org/10.1038/s41434-019-0078-3.

8. Ewald, A.J., Huebner, R.J., Palsdottir, H., Lee, J.K., Perez, M.J., Jorgens, D.M., Tauscher, A.N., Cheung, K.J., Werb, Z., and Auer, M. (2012). Mammary collective cell migration involves transient loss of epithelial features and individual cell migration within the epithelium. J. Cell Sci., jcs.096875. https://doi.org/10.1242/jcs.096875.

9. Xu, C.S., Hayworth, K.J., Lu, Z., Grob, P., Hassan, A.M., García-Cerdán, J.G., Niyogi, K.K., Nogales, E., Weinberg, R.J., and Hess, H.F. (2017). Enhanced FIB-SEM systems for large-volume 3D imaging. eLife *6*, e25916. https://doi.org/10.7554/eLife.25916.

10. Xu, C.S., Pang, S., Shtengel, G., Müller, A., Ritter, A.T., Hoffman, H.K., Takemura, S., Lu, Z., Pasolli, H.A., Iyer, N., et al. (2021). An open-access volume electron microscopy atlas of whole cells and tissues. Nature *599*, 147–151. https://doi.org/10.1038/s41586-021-03992-4.

11. Mudge, J.M., Carbonell-Sala, S., Diekhans, M., Martinez, J.G., Hunt, T., Jungreis, I., Loveland, J.E., Arnan, C., Barnes, I., Bennett, R., et al. (2025). GENCODE 2025: reference gene annotation for human and mouse. Nucleic Acids Res. *53*, D966–D975.

bioRxiv preprint doi: https://doi.org/10.64898/2026.08.30.747939; this version posted September 1, 2026. The copyright holder for this preprint (which was not certified by peer review) is the author/funder, who has granted bioRxiv a license to display the preprint in perpetuity. It is made available under a CC-BY 4.0 International license.

https://doi.org/10.1093/nar/gkae1078.

12. Robinson, M.D., McCarthy, D.J., and Smyth, G.K. (2010). edgeR : a Bioconductor package for differential expression analysis of digital gene expression data. Bioinformatics *26*, 139–140. https://doi.org/10.1093/bioinformatics/btp616.

13. Kolde, R. (2025). *pheatmap: Pretty Heatmaps* (R package version 1.0.13), Github. https://github.com/raivokolde/pheatmap.

14. Huang, D.W., Sherman, B.T., and Lempicki, R.A. (2009). Bioinformatics enrichment tools: paths toward the comprehensive functional analysis of large gene lists. Nucleic Acids Res. *37*, 1–13. https://doi.org/10.1093/nar/gkn92

bioRxiv preprint doi: https://doi.org/10.64898/2026.08.30.747939; this version posted September 1, 2026. The copyright holder for this preprint (which was not certified by peer review) is the author/funder, who has granted bioRxiv a license to display the preprint in perpetuity. It is made available under a CC-BY 4.0 International license.

Supplementary Figure S1

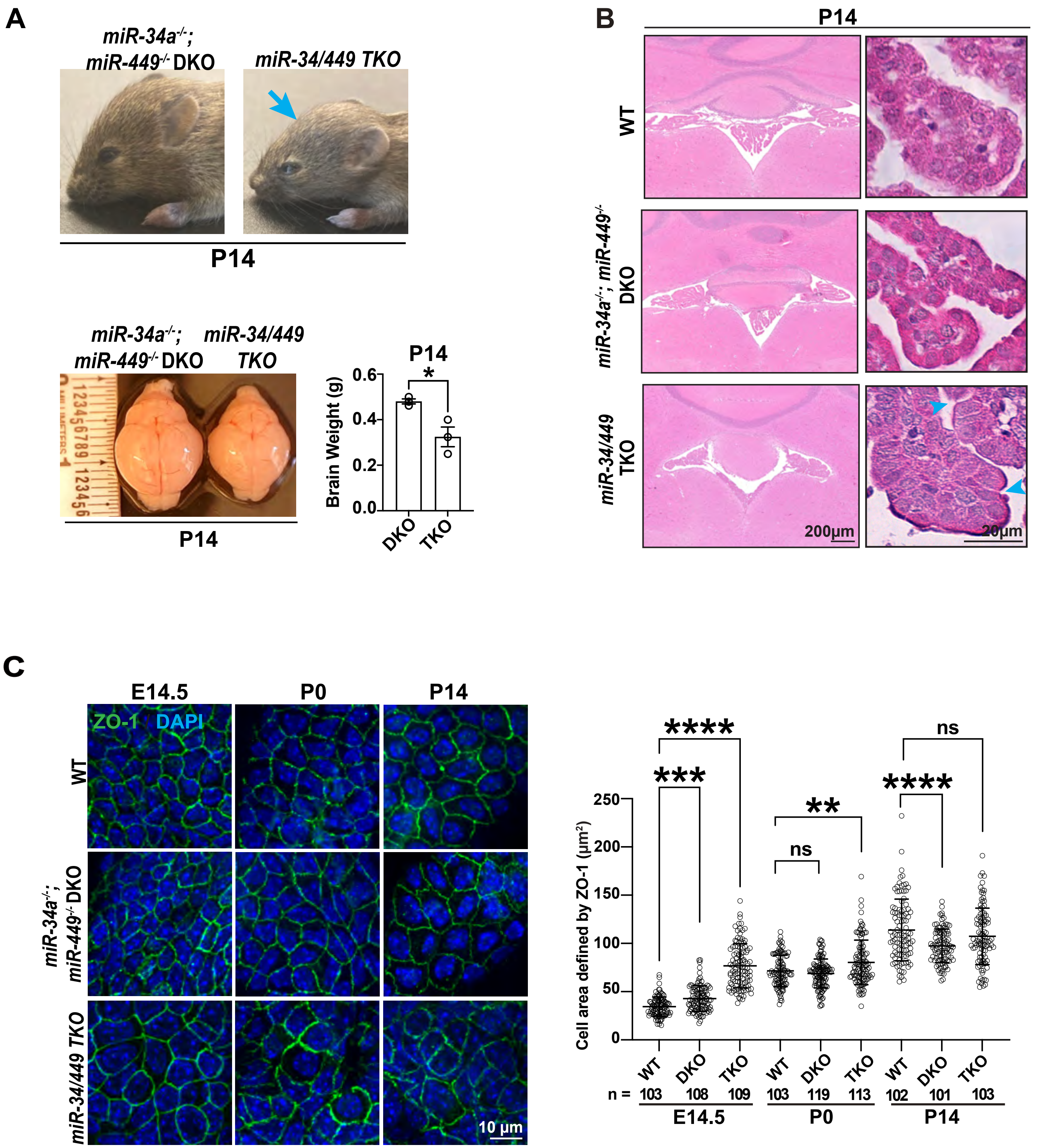

bioRxiv preprint doi: https://doi.org/10.64898/2026.08.30.747939; this version posted September 1, 2026. The copyright holder for this preprint (which was not certified by peer review) is the author/funder, who has granted bioRxiv a license to display the preprint in perpetuity. It is made available under a CC-BY 4.0 International license.

Supplementary Figure S1

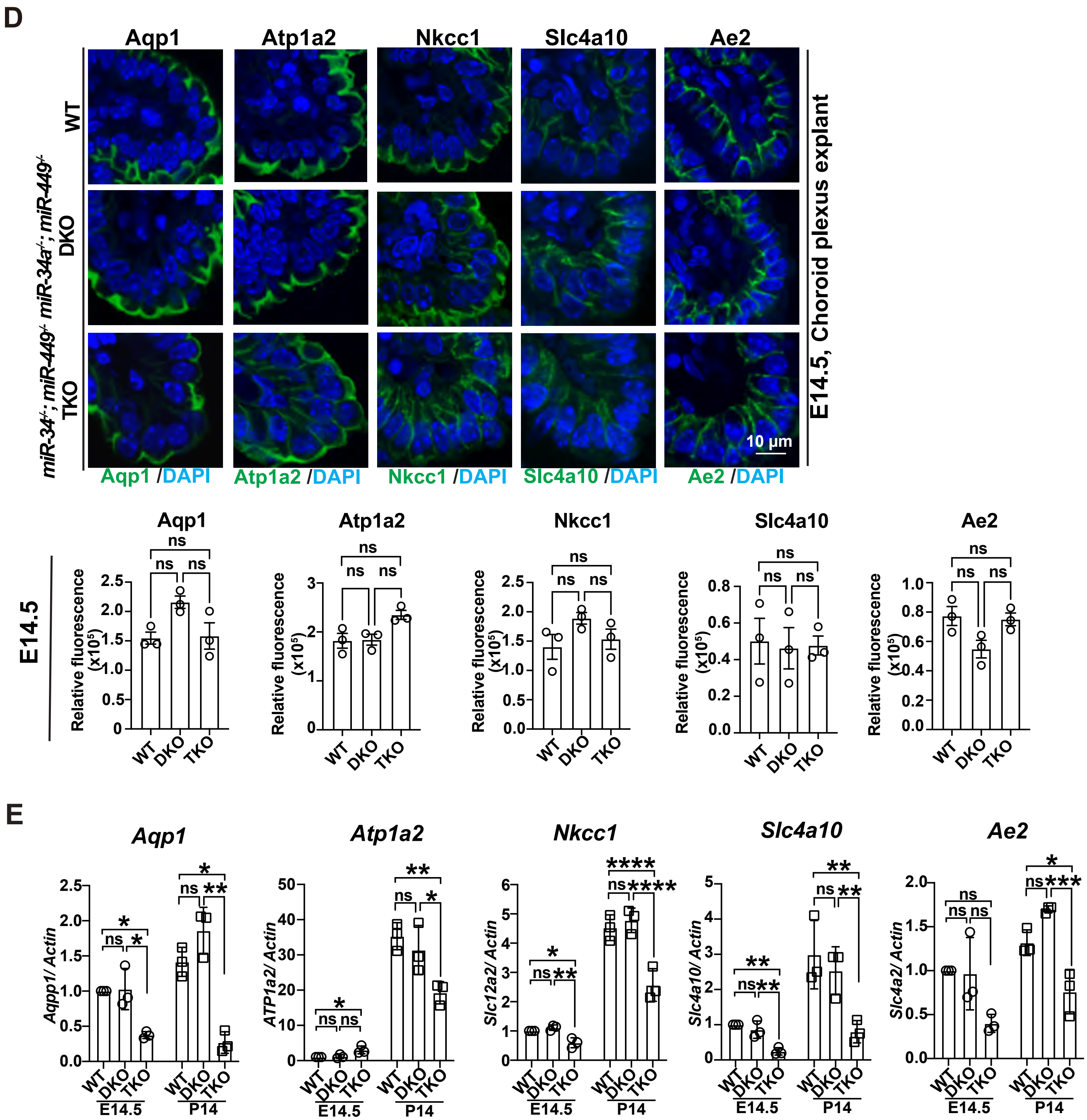

bioRxiv preprint doi: https://doi.org/10.64898/2026.08.30.747939; this version posted September 1, 2026. The copyright holder for this preprint (which was not certified by peer review) is the author/funder, who has granted bioRxiv a license to display the preprint in perpetuity. It is made available under a CC-BY 4.0 International license.

Supplementary Figure S2

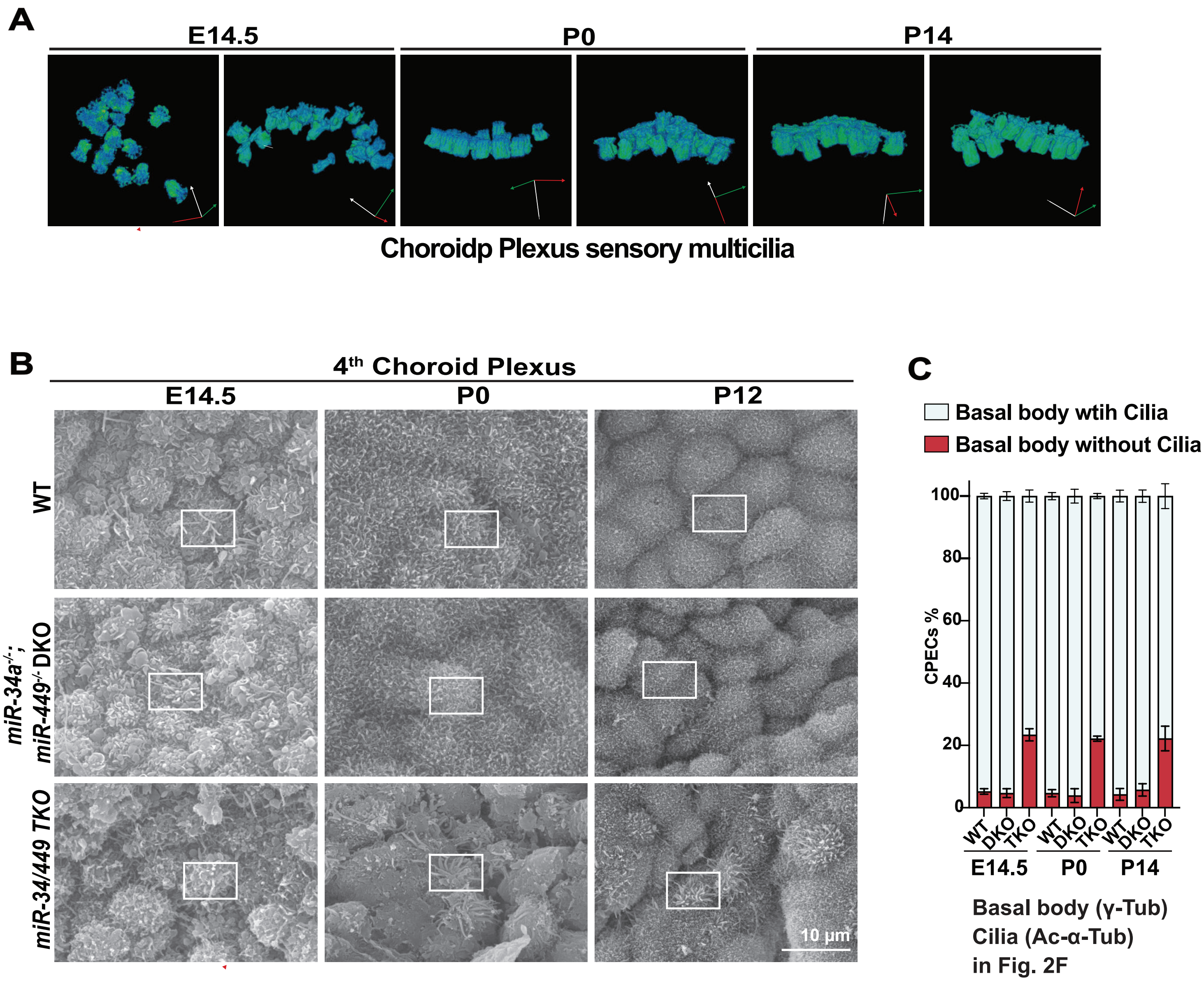

bioRxiv preprint doi: https://doi.org/10.64898/2026.08.30.747939; this version posted September 1, 2026. The copyright holder for this preprint (which was not certified by peer review) is the author/funder, who has granted bioRxiv a license to display the preprint in perpetuity. It is made available under a CC-BY 4.0 International license.

Supplementary Figure S3

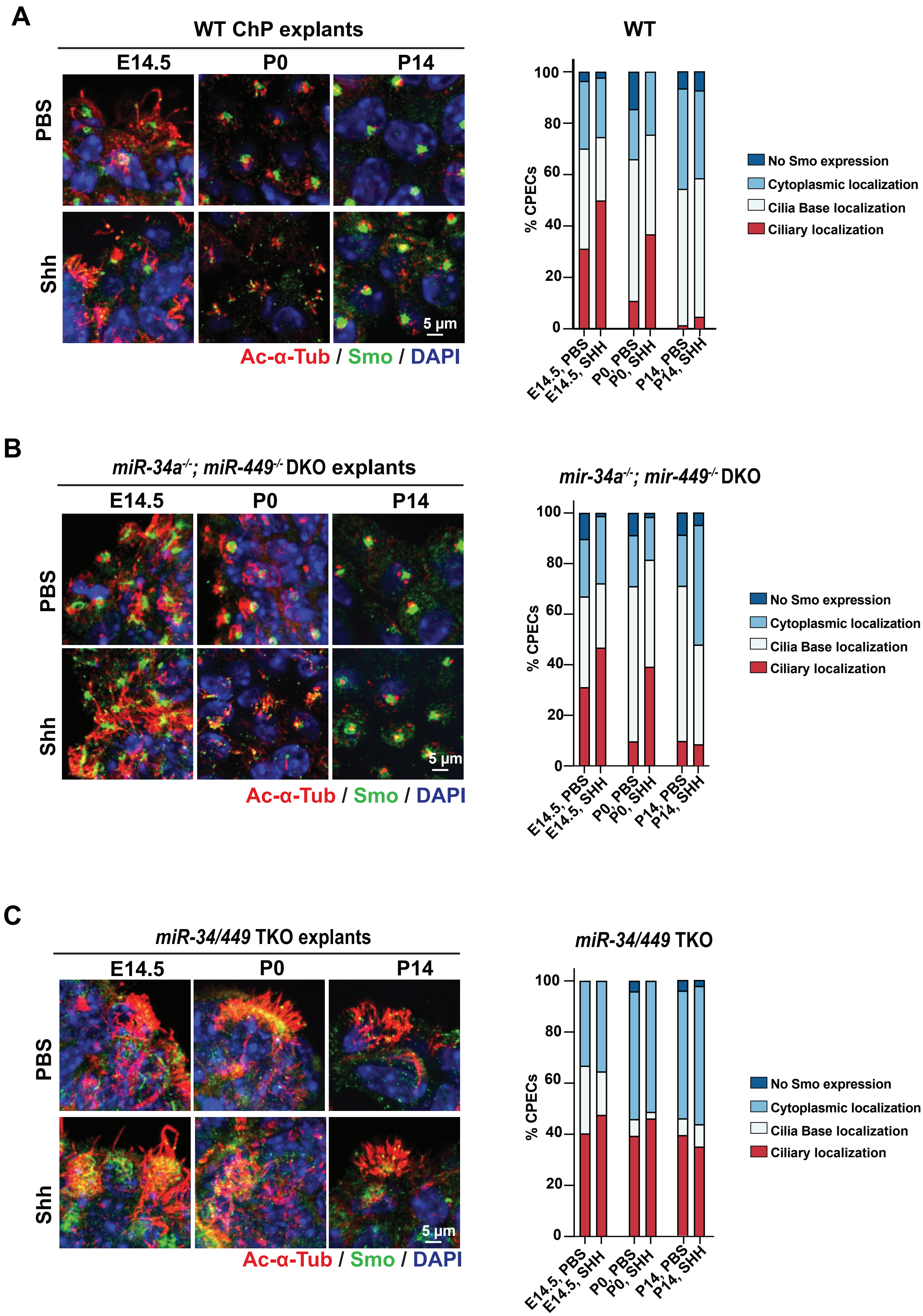

bioRxiv preprint doi: https://doi.org/10.64898/2026.08.30.747939; this version posted September 1, 2026. The copyright holder for this preprint (which was not certified by peer review) is the author/funder, who has granted bioRxiv a license to display the preprint in perpetuity. It is made available under a CC-BY 4.0 International license.

Supplementary Figure S3

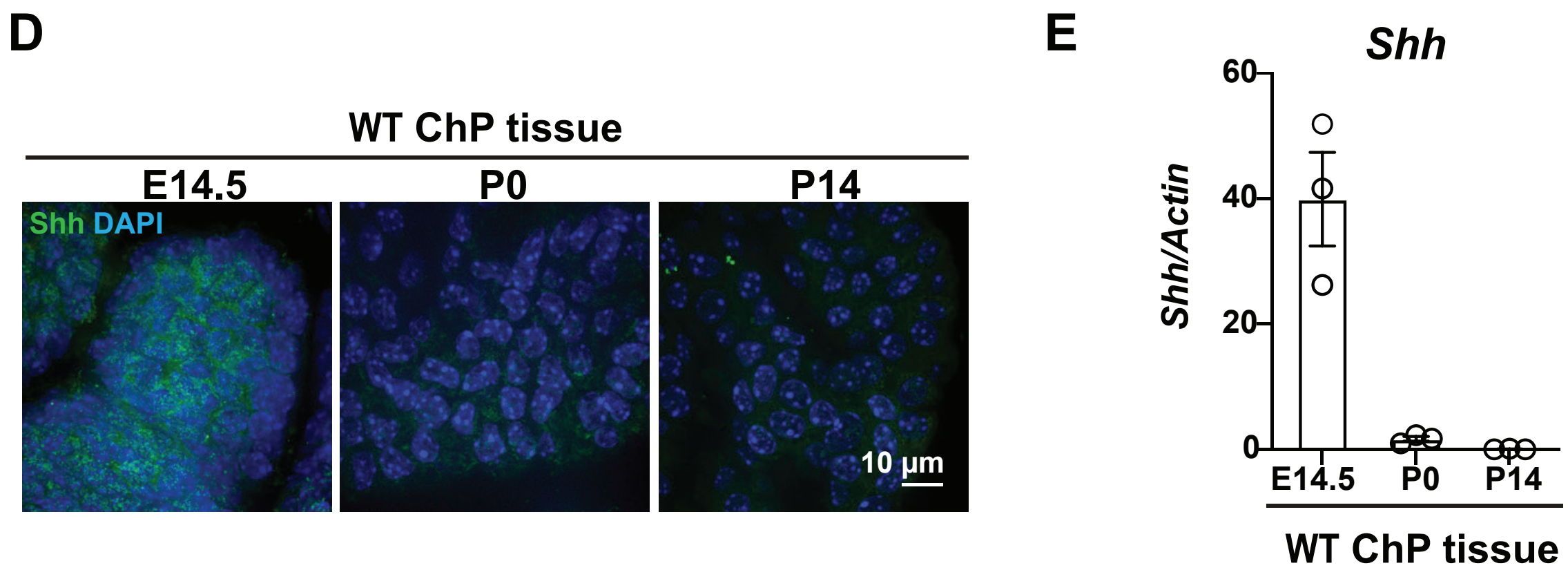

bioRxiv preprint doi: https://doi.org/10.64898/2026.08.30.747939; this version posted September 1, 2026. The copyright holder for this preprint (which was not certified by peer review) is the author/funder, who has granted bioRxiv a license to display the preprint in perpetuity. It is made available under a CC-BY 4.0 International license.

Supplementary Figure S4

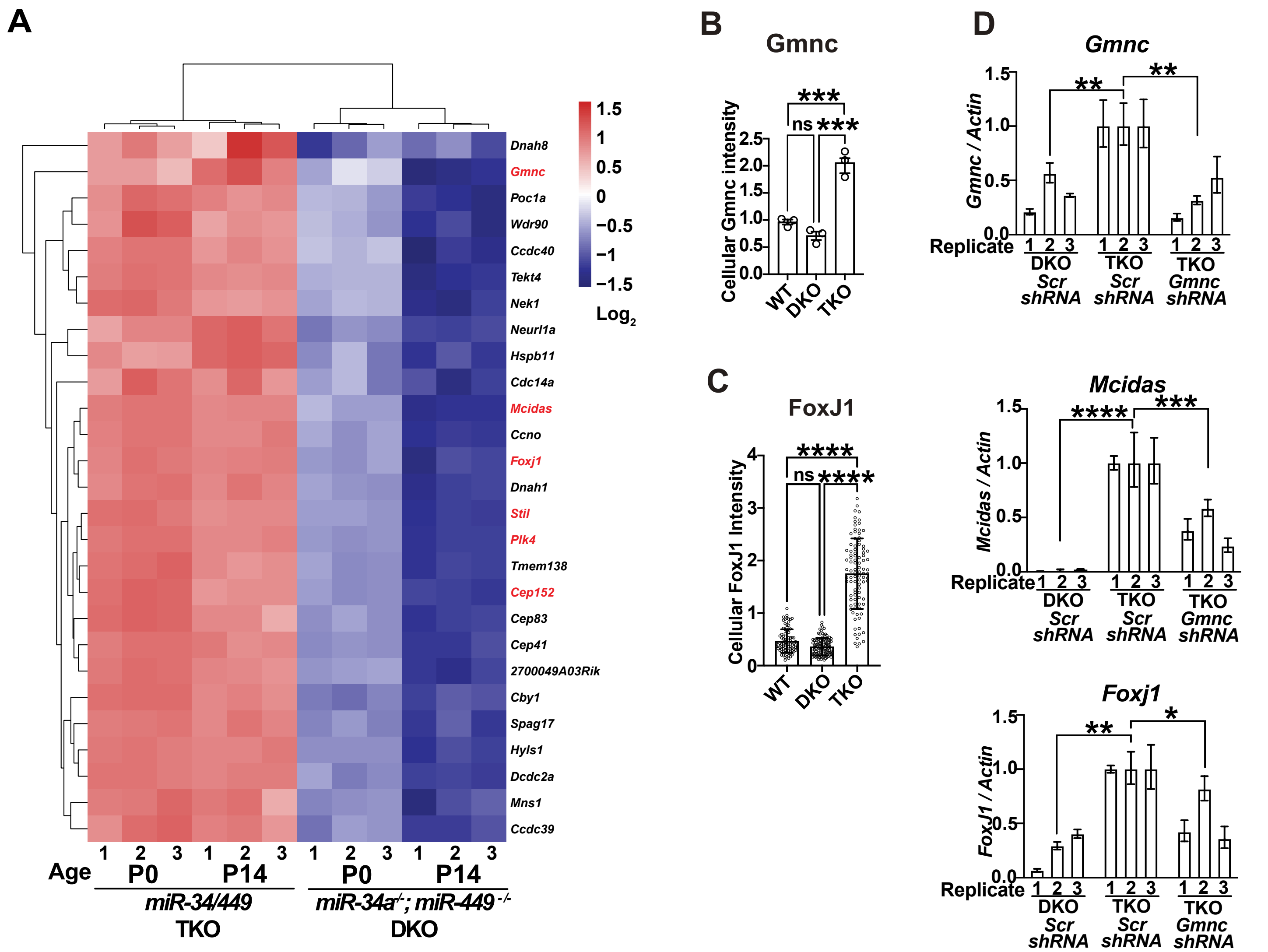

bioRxiv preprint doi: https://doi.org/10.64898/2026.08.30.747939; this version posted September 1, 2026. The copyright holder for this preprint (which was not certified by peer review) is the author/funder, who has granted bioRxiv a license to display the preprint in perpetuity. It is made available under a CC-BY 4.0 International license.

Table S1. Top 100 dynamically expressed genes in P0 and p14 DKO and TKO choroid plexus, related to Figure 4.

Gene	ave.P0 DKO	ave.P0 TKO	ave.P14 DKO	ave.P14 TKO	logFC.p0	logCPM.p0	FDR.p0	logFC.p14	logCPM.p14	FDR.p14
Ccna1	5.74	266.47	0.99	298.53	5.39	6.47	2.00E-12	7.53	6.47	3.84E-13
E2f7	6.50	184.15	2.66	124.17	4.23	7.44	2.00E-12	5.16	7.44	3.84E-13
Dnaaf1	3.95	67.33	2.23	62.05	3.72	5.95	4.51E-12	4.04	5.95	1.84E-12
Elof1	86.99	546.12	70.80	506.09	2.20	7.08	4.51E-12	1.93	7.08	5.50E-12
Foxn4	1.47	69.78	0.18	57.90	5.38	6.00	4.51E-12	7.96	6.00	1.27E-12
Myb	10.42	215.27	2.19	209.07	4.10	7.44	4.51E-12	5.71	7.44	5.30E-13
Tedc1	1.30	43.66	0.46	48.01	4.73	4.97	4.51E-12	5.15	4.97	1.27E-12
Tppp3	38.91	625.46	16.47	673.10	3.60	7.73	4.51E-12	4.58	7.73	5.30E-13
Cdhr4	3.65	114.13	3.61	159.45	4.96	5.65	4.86E-12	5.16	5.65	1.84E-12
Poc5	11.81	95.04	7.74	83.59	2.56	6.58	4.86E-12	2.55	6.58	2.95E-12
Sord	29.08	180.02	23.01	128.68	2.28	7.02	4.86E-12	1.74	7.02	1.58E-11
Stil	4.74	110.64	0.84	77.61	4.14	6.80	5.64E-12	5.58	6.80	1.27E-12
Cdhr3	2.12	130.80	1.66	161.63	5.63	6.76	6.60E-12	5.86	6.76	2.95E-12
Rsph1	79.51	630.33	103.25	634.37	2.63	7.81	7.56E-12	1.88	7.81	3.52E-11
1700003E	6.44	114.41	5.96	103.25	3.79	6.36	8.04E-12	3.36	6.36	9.16E-12
Acox2	6.32	54.55	20.70	70.33	3.10	5.63	9.58E-12	0.91	5.63	8.94E-08
Dnai2	17.89	164.22	14.31	139.23	3.00	7.10	9.58E-12	2.62	7.10	1.29E-11
Efhc1	43.57	220.57	32.44	197.98	2.05	7.02	9.58E-12	2.08	7.02	6.53E-12
Hdc	35.68	351.99	27.81	411.59	2.89	7.46	9.58E-12	3.14	7.46	3.63E-12
Lrrc46	19.13	233.00	7.50	199.67	3.24	6.15	9.58E-12	3.99	6.15	2.85E-12
Lrwd1	12.34	133.65	5.28	105.14	3.04	6.77	9.58E-12	3.55	6.77	2.88E-12
Ruvbl2	36.09	337.02	17.78	257.68	2.88	7.35	9.58E-12	3.09	7.35	4.21E-12
Stk33	11.77	120.66	2.16	87.54	3.37	5.76	9.58E-12	4.90	5.76	1.65E-12
Syt10	1.67	132.72	0.50	95.07	5.97	5.66	9.58E-12	6.82	5.66	7.12E-12
Tekt4	17.22	125.85	4.64	103.58	2.46	5.61	1.12E-11	3.72	5.61	1.27E-12
Kif19a	2.11	30.80	1.05	36.86	3.95	5.20	1.22E-11	4.35	5.20	5.50E-12
Sostdc1	720.32	124.09	786.20	204.24	-2.91	9.11	1.22E-11	-2.68	9.11	1.10E-11
Iqca	8.27	72.94	2.87	75.77	2.70	5.97	1.42E-11	3.88	5.97	1.41E-12
Ccdc153	3.02	461.10	10.47	608.72	6.71	6.32	1.56E-11	5.03	6.32	1.99E-11
Cfap161	5.03	88.61	4.52	93.90	3.76	5.05	1.56E-11	3.60	5.05	1.10E-11
Dzank1	5.12	45.76	5.85	41.39	2.60	6.45	1.56E-11	2.21	6.45	2.67E-11
Plk4	12.24	293.12	3.72	220.73	3.95	7.01	1.56E-11	4.65	7.01	5.66E-12
Ccdc146	15.99	131.19	16.09	132.62	2.97	6.57	1.71E-11	2.80	6.57	1.44E-11
Gm28729	5.53	67.49	9.56	58.46	3.24	5.30	1.79E-11	1.86	5.30	5.31E-10
Dpy19l2	1.50	42.01	0.49	33.65	4.43	4.94	1.80E-11	5.32	4.94	8.13E-12
Cep76	7.48	48.48	5.04	35.93	2.44	5.46	1.86E-11	2.17	5.46	2.83E-11
Gm9899	0.26	18.37	0.17	16.24	5.86	4.81	1.86E-11	7.31	4.81	9.20E-12
Tmem107	65.38	598.45	54.99	557.55	2.57	6.43	1.86E-11	2.48	6.43	1.50E-11
Deup1	24.52	520.25	3.73	393.83	3.99	8.01	1.86E-11	5.81	8.01	1.94E-12
Drc3	42.89	218.97	36.20	214.19	1.97	7.00	1.86E-11	1.81	7.00	2.29E-11
Gm30239	1.65	100.32	0.38	91.70	5.58	3.60	1.86E-11	7.14	3.60	1.00E-11
Lrrc23	225.77	1,255.49	90.30	1,210.01	2.45	8.10	1.86E-11	3.34	8.10	1.88E-12
Scn4b	0.39	30.45	0.14	24.14	5.91	5.74	1.86E-11	6.70	5.74	1.26E-11
Shisa8	2.16	80.23	0.79	63.97	4.62	5.95	1.86E-11	5.71	5.95	8.25E-12
Vat1l	787.14	193.64	692.44	191.25	-2.43	10.36	1.86E-11	-2.72	10.36	6.60E-12
BC051019	4.37	94.64	1.15	89.51	4.18	5.46	2.06E-11	5.86	5.46	5.52E-12
Mcidas	7.00	149.23	0.79	116.64	4.04	6.59	2.06E-11	6.47	6.59	2.88E-12
Osbpl6	3.70	34.57	1.38	51.68	2.87	5.78	2.06E-11	4.47	5.78	1.38E-12
Ttll9	14.12	162.18	11.03	106.46	3.16	5.88	2.08E-11	2.72	5.88	4.68E-11
1700016K	33.80	354.30	21.71	326.50	3.00	6.08	2.15E-11	3.19	6.08	1.05E-11

bioRxiv preprint doi: https://doi.org/10.64898/2026.08.30.747939; this version posted September 1, 2026. The copyright holder for this preprint (which was not certified by peer review) is the author/funder, who has granted bioRxiv a license to display the preprint in perpetuity. It is made available under a CC-BY 4.0 International license.

4921536K	4.19	31.84	2.48	29.11	2.59	4.88	2.20E-11	2.81	4.88	1.01E-11
Ccdc121	0.37	31.59	0.63	40.79	5.99	4.03	2.20E-11	5.17	4.03	2.00E-11
Lrguk	7.53	62.74	4.12	52.49	2.65	5.86	2.20E-11	2.74	5.86	1.44E-11
Map3k19	8.70	64.03	6.30	56.08	2.50	6.71	2.20E-11	2.37	6.71	2.25E-11
Ccdc78	0.34	55.17	0.54	81.96	6.92	5.07	2.47E-11	6.40	5.07	1.50E-11
Cep135	6.94	51.81	4.60	37.07	2.52	6.05	2.47E-11	2.26	6.05	4.18E-11
Crip3	13.72	633.75	6.36	707.89	5.15	6.64	2.51E-11	6.51	6.64	8.25E-12
Diras2	0.84	18.58	0.95	20.87	4.12	5.03	2.51E-11	3.73	5.03	2.78E-11
2900040C	486.03	71.81	348.18	24.98	-3.09	5.66	2.73E-11	-4.24	5.66	5.50E-12
Ldhb	450.68	94.53	942.74	185.77	-2.61	8.65	2.89E-11	-3.06	8.65	7.40E-12
Cav2	20.52	131.38	11.81	126.53	2.33	6.27	2.97E-11	2.70	6.27	8.25E-12
Ak9	9.48	57.71	7.38	48.17	2.26	6.79	3.13E-11	1.98	6.79	5.80E-11
Dcdc2a	41.70	387.44	21.19	350.11	2.65	8.39	3.13E-11	3.54	8.39	3.63E-12
Ipmk	7.65	31.54	4.81	32.34	1.69	5.69	3.13E-11	2.05	5.69	7.12E-12
Dnah5	0.35	22.44	0.52	22.18	5.64	6.68	3.22E-11	4.65	6.68	5.62E-11
Hyls1	6.88	108.28	2.56	100.87	3.54	5.96	3.33E-11	4.46	5.96	8.25E-12
Morn5	15.68	179.85	16.25	216.42	3.15	4.92	3.33E-11	3.02	4.92	2.67E-11
Thsd7b	17.71	2.01	16.37	2.28	-3.46	5.29	3.33E-11	-3.82	5.29	1.10E-11
Tmem67	18.19	96.77	10.82	76.76	2.04	6.50	3.33E-11	2.10	6.50	2.10E-11
Zswim2	0.57	28.92	0.07	22.61	5.41	3.25	3.33E-11	7.44	3.25	2.03E-11
Ankrd42	15.14	64.83	9.92	57.41	1.78	5.87	3.58E-11	1.84	5.87	2.14E-11
Spag17	5.08	82.35	2.05	74.55	3.99	6.97	3.70E-11	4.95	6.97	9.60E-12
1700012B	51.52	742.99	16.46	729.33	3.52	6.14	4.06E-11	4.74	6.14	7.83E-12
Gm11992	12.78	58.52	14.61	60.49	1.82	5.50	4.06E-11	1.27	5.50	4.37E-10
Traf3ip1	13.92	89.22	12.99	82.53	2.31	6.51	4.06E-11	1.91	6.51	1.12E-10
1700001L	9.13	170.81	2.66	165.19	3.84	5.03	4.23E-11	5.07	5.03	9.75E-12
Ect2	6.04	62.44	3.22	51.27	3.09	5.70	4.23E-11	3.31	5.70	2.45E-11
Atp7a	103.16	25.59	80.63	20.55	-2.39	7.63	4.39E-11	-2.82	7.63	9.64E-12
6820408C	29.97	211.57	14.31	192.52	2.43	6.09	4.40E-11	2.91	6.09	1.00E-11
Ccdc170	19.25	78.29	11.75	77.31	1.67	6.21	4.67E-11	2.04	6.21	8.25E-12
Cep41	33.99	136.65	22.19	121.80	1.58	6.50	4.67E-11	1.63	6.50	2.48E-11
Nt5dc3	3.06	32.33	3.69	27.59	2.99	6.13	4.71E-11	2.15	6.13	3.64E-10
Saxo2	46.10	262.67	34.60	215.90	2.71	6.23	4.76E-11	2.93	6.23	2.14E-11
Morn3	9.17	95.94	7.40	128.76	3.07	4.89	4.85E-11	3.26	4.89	2.06E-11
Stmnd1	0.45	46.61	0.33	45.67	6.32	3.69	5.27E-11	6.34	3.69	4.30E-11
Sema3b	148.15	30.84	83.34	36.22	-2.66	7.52	5.46E-11	-1.97	7.52	3.44E-10
Ccno	8.43	235.32	1.32	195.95	4.37	7.04	5.53E-11	6.43	7.04	8.25E-12
Zfp474	3.81	110.74	2.20	124.85	4.29	5.58	5.53E-11	4.68	5.58	2.47E-11
2610028H	0.62	90.95	0.30	151.45	6.86	5.81	5.59E-11	8.31	5.81	1.58E-11
Ak8	19.28	106.75	12.21	88.90	2.16	5.59	5.61E-11	2.18	5.59	4.14E-11
Elmod1	14.35	148.65	5.66	125.89	3.07	6.39	5.61E-11	3.69	6.39	1.44E-11
Dnah7c	0.96	11.66	0.56	11.89	3.35	5.32	5.76E-11	3.59	5.32	2.75E-11
Plb1	0.23	16.38	0.18	8.48	6.23	3.99	5.76E-11	6.11	3.99	1.39E-10
Nat9	7.89	89.62	5.40	97.44	2.94	4.56	6.03E-11	3.11	4.56	2.83E-11
Fxyd1	972.79	132.45	1,183.34	244.96	-3.30	7.22	6.40E-11	-2.86	7.22	1.01E-10
Dpp6	1.71	38.73	1.29	42.22	4.27	5.86	6.42E-11	4.29	5.86	4.16E-11
2510009E	25.61	10.43	22.38	12.60	-1.67	6.00	6.95E-11	-1.62	6.00	5.32E-11
Cep43	26.15	155.76	15.60	114.06	2.19	6.69	6.95E-11	2.10	6.69	6.89E-11
Ruvbl1	34.28	152.81	23.59	124.79	1.80	6.35	6.95E-11	1.66	6.35	9.42E-11
Cep152	5.07	48.17	2.17	33.57	2.86	6.13	6.99E-11	3.19	6.13	2.83E-11
Dnai3	36.30	247.85	21.37	203.99	2.47	7.49	7.06E-11	2.51	7.49	4.21E-11

bioRxiv preprint doi: https://doi.org/10.64898/2026.08.30.747939; this version posted September 1, 2026. The copyright holder for this preprint (which was not certified by peer review) is the author/funder, who has granted bioRxiv a license to display the preprint in perpetuity. It is made available under a CC-BY 4.0 International license.

Table S2. Upregulated genes that are predicted miR-34/449-targets

Gene	Description
Elmod1	ELMO/CED-12 domain containing 1
Usp31	ubiquitin specific peptidase 31
Jag1	jagged 1
Hk1	hexokinase 1
Lhfpl4	lipoma HMGIC fusion partner-like protein 4
Arhgap1	Rho GTPase activating protein 1
Scml2	Scm polycomb group protein like 2
Myrip	myosin VIIA and Rab interacting protein
Ldlrad2	low density lipoprotein receptor class A domain containing 2
Fam167a	family with sequence similarity 167, member A
Ralgds	ral guanine nucleotide dissociation stimulator
Slc6a17	solute carrier family 6 (neurotransmitter transporter), member 17
Rdh11	retinol dehydrogenase 11
Rai14	retinoic acid induced 14
Cybrd1	cytochrome b reductase 1
Shisa7	shisa family member 7
Elovl6	ELOVL fatty acid elongase 6
Pdk2	pyruvate dehydrogenase kinase, isoenzyme 2
Dixdc1	DIX domain containing 1
Frmd4a	FERM domain containing 4A
Map1a	microtubule-associated protein 1 A
Fam76a	family with sequence similarity 76, member A
Fut9	fucosyltransferase 9
Arhgap39	Rho GTPase activating protein 39
Adam22	a disintegrin and metallopeptidase domain 22
Trpm3	transient receptor potential cation channel, subfamily M, member 3
Ahcyl2	S-adenosylhomocysteine hydrolase-like 2
Ltbp2	latent transforming growth factor beta binding protein 2
Adam10	a disintegrin and metallopeptidase domain 10
Acsl4	acyl-CoA synthetase long-chain family member 4
Rtn4rl1	reticulon 4 receptor-like 1
Zic5	zinc finger protein of the cerebellum 5
Tmem231	transmembrane protein 231
Bcl2	B cell leukemia/lymphoma 2
Lef1	lymphoid enhancer binding factor 1
Notch1	notch 1
Cplx2	complexin 2
Slc25a23	solute carrier family 25 (mitochondrial carrier; phosphate carrier), member 23
Btbd11	BTB domain containing 11
Golph3l	golgi phosphoprotein 3-like
Thra	thyroid hormone receptor alpha

bioRxiv preprint doi: https://doi.org/10.64898/2026.08.30.747939; this version posted September 1, 2026. The copyright holder for this preprint (which was not certified by peer review) is the author/funder, who has granted bioRxiv a license to display the preprint in perpetuity. It is made available under a CC-BY 4.0 International license.

Nceh1	neutral cholesterol ester hydrolase 1
Tert	telomerase reverse transcriptase
Tpd52	tumor protein D52
Cpeb2	cytoplasmic polyadenylation element binding protein 2
Htr2c	5-hydroxytryptamine (serotonin) receptor 2C
Pitpnc1	phosphatidylinositol transfer protein, cytoplasmic 1
Atmin	ATM interactor
Acsl1	acyl-CoA synthetase long-chain family member 1
Usp24	ubiquitin specific peptidase 24
Pip5k1a	phosphatidylinositol-4-phosphate 5-kinase, type 1 alpha
Ankfy1	ankyrin repeat and FYVE domain containing 1
Tmcc3	transmembrane and coiled coil domains 3
Tanc2	tetratricopeptide repeat, ankyrin repeat and coiled-coil containing 2
Pkp4	plakophilin 4
Ppp1r11	protein phosphatase 1, regulatory inhibitor subunit 11
Fam83h	family with sequence similarity 83, member H
Cnot6	CCR4-NOT transcription complex, subunit 6
Jmjd1c	jumonji domain containing 1C
Sdhc	succinate dehydrogenase complex, subunit C, integral membrane protein
Chd1	chromodomain helicase DNA binding protein 1
Grhl2	grainyhead like transcription factor 2
Ralgps2	Ral GEF with PH domain and SH3 binding motif 2
Tm7sf3	transmembrane 7 superfamily member 3
Pan3	PAN3 poly(A) specific ribonuclease subunit
Rnf152	ring finger protein 152
Cntnap2	contactin associated protein-like 2
Dgkz	diacylglycerol kinase zeta
Arid4a	AT-rich interaction domain 4A
Kcnk3	potassium channel, subfamily K, member 3
Synj1	synaptojanin 1
Ppp2r3a	protein phosphatase 2, regulatory subunit B'', alpha
Magi2	membrane associated guanylate kinase, WW and PDZ domain containing 2
Tmem251	Transmembrane protein 251
Lonrf3	LON peptidase N-terminal domain and ring finger 3
Brpf3	bromodomain and PHD finger containing, 3
Ap1s2	adaptor-related protein complex 1, sigma 2 subunit
Lin54	lin-54 DREAM MuvB core complex component
Gfra1	glial cell line derived neurotrophic factor family receptor alpha 1
Rab43	RAB43, member RAS oncogene family
Ralgps1	Ral GEF with PH domain and SH3 binding motif 1
Tmed8	transmembrane p24 trafficking protein 8
Mpp2	membrane protein, palmitoylated 2 (MAGUK p55 subfamily member 2)
Grid1	glutamate receptor, ionotropic, delta 1

bioRxiv preprint doi: https://doi.org/10.64898/2026.08.30.747939; this version posted September 1, 2026. The copyright holder for this preprint (which was not certified by peer review) is the author/funder, who has granted bioRxiv a license to display the preprint in perpetuity. It is made available under a CC-BY 4.0 International license.

Rragd	Ras-related GTP binding D
Shank3	SH3 and multiple ankyrin repeat domains 3
Atxn7	ataxin 7
Atp2b4	ATPase, Ca++ transporting, plasma membrane 4
Aldoa	aldolase A, fructose-bisphosphate
Cacna2d1	calcium channel, voltage-dependent, alpha2/delta subunit 1
Naa50	N(alpha)-acetyltransferase 50, NatE catalytic subunit
Taf4b	TATA-box binding protein associated factor 4b
Sptbn2	spectrin beta, non-erythrocytic 2
Mras	muscle and microspikes RAS
Gjb4	gap junction protein, beta 4
Ret	ret proto-oncogene
E2f5	E2F transcription factor 5
Dpysl4	dihydropyrimidinase-like 4
Limd2	LIM domain containing 2
Cdh4	cadherin 4
Slc35g2	solute carrier family 35, member G2
Pax5	paired box 5
C1ql3	C1q-like 3
Gnai2	G protein subunit alpha i2
Fndc3b	fibronectin type III domain containing 3B
Agap2	ArfGAP with GTPase domain, ankyrin repeat and PH domain 2
Pomgnt1	protein O-linked mannose beta 1,2-N-acetylglucosaminyltransferase
Tmem126b	transmembrane protein 126B
Ikzf1	IKAROS family zinc finger 1
Ap5m1	adaptor-related protein complex 5, mu 1 subunit
Srr	serine racemase
Rab11fip4	RAB11 family interacting protein 4 (class II)
Tbc1d25	TBC1 domain family, member 25
Sidt1	SID1 transmembrane family, member 1
Ndrg4	N-myc downstream regulated gene 4
Tab1	TGF-beta activated kinase 1/MAP3K7 binding protein 1
Snx30	sorting nexin family member 30
Mapk1	mitogen-activated protein kinase 1
Dmwd	dystrophia myotonica-containing WD repeat motif
Creld1	cysteine-rich with EGF-like domains 1
Srpr	Signal Recognition Particle (SRP) receptor
Plekhg3	Pleckstrin Homology and RhoGEF Domain Containing G3
Pdgfra	platelet derived growth factor receptor, alpha polypeptide
Eea1	early endosome antigen 1
Uhrf2	ubiquitin-like, containing PHD and RING finger domains 2
Dagla	diacylglycerol lipase, alpha
Tbc1d30	TBC1 domain family, member 30

bioRxiv preprint doi: https://doi.org/10.64898/2026.08.30.747939; this version posted September 1, 2026. The copyright holder for this preprint (which was not certified by peer review) is the author/funder, who has granted bioRxiv a license to display the preprint in perpetuity. It is made available under a CC-BY 4.0 International license.

Sema4b	sema domain (semaphorin) 4B
Wdr37	WD repeat domain 37
Asphd2	aspartate beta-hydroxylase domain containing 2
Raly	hnRNP-associated with lethal yellow
Inhbb	inhibin beta-B
Maz	MYC-associated zinc finger protein (purine-binding transcription factor)
Bcl11b	B cell leukemia/lymphoma 11B
Foxp1	forkhead box P1
Abr	active BCR-related gene
Cntd1	cyclin N-terminal domain containing 1
Gnaq	guanine nucleotide binding protein, alpha q polypeptide
Asic2	acid-sensing ion channel 2
Sema4f	sema domain (semaphorin) 4F
Dgkh	diacylglycerol kinase, eta
Ppwd1	peptidylprolyl isomerase domain and WD repeat containing 1
Tfdp2	transcription factor Dp 2
Reln	reelin
Ttpal	tocopherol (alpha) transfer protein-like
Oxsr1	oxidative-stress responsive 1
Mtus1	mitochondrial tumor suppressor 1
Galnt7	polypeptide N-acetylgalactosaminyltransferase 7
Satb2	special AT-rich sequence binding protein 2
Mcfd2	multiple coagulation factor deficiency 2
Ttc19	tetratricopeptide repeat domain 19
Foxp2	forkhead box P2
Pacs1	phosphofurin acidic cluster sorting protein 1
Cntnap1	contactin associated protein-like 1
Wasf1	WASP family, member 1
Casp2	caspase 2
Gabrb1	gamma-aminobutyric acid type A receptor subunit beta 1
Arhgap36	Rho GTPase activating protein 36
Gabra4	gamma-aminobutyric acid type A receptor subunit alpha 4
Ank3	ankyrin 3, epithelial
Fgd6	FYVE, RhoGEF and PH domain containing 6
Mta2	metastasis-associated gene family, member 2
Six3	sine oculis-related homeobox 3
Ythdc1	YTH domain containing 1
Eif2s2	eukaryotic translation initiation factor 2 subunit 2 beta
Crtc1	CREB regulated transcription coactivator 1
Capn6	calpain 6
Syt1	synaptotagmin I
Igfbp3	insulin-like growth factor binding protein 3
Unc13c	unc-13 homolog C

bioRxiv preprint doi: https://doi.org/10.64898/2026.08.30.747939; this version posted September 1, 2026. The copyright holder for this preprint (which was not certified by peer review) is the author/funder, who has granted bioRxiv a license to display the preprint in perpetuity. It is made available under a CC-BY 4.0 International license.

Rps6ka4	ribosomal protein S6 kinase, polypeptide 4
Hcn3	hyperpolarization-activated, cyclic nucleotide-gated K+ 3
Cacnb3	calcium channel, voltage-dependent, beta 3 subunit
Capn5	calpain 5
Lhx2	LIM homeobox protein 2
Smc6	structural maintenance of chromosomes 6
Add2	adducin 2
Rnf213	ring finger protein 213
Brinp1	bone morphogenic protein/retinoic acid inducible neural specific 1
Csf1r	colony stimulating factor 1 receptor
Ern1	endoplasmic reticulum to nucleus signalling 1
Rras	related RAS viral (r-ras) oncogene
Cbfa2t3	CBFA2/RUNX1 translocation partner 3
Lman1	lectin, mannose-binding, 1
Nrn1	neuritin 1
Cacna1e	calcium channel, voltage-dependent, R type, alpha 1E subunit
Foxn2	forkhead box N2
Grm7	glutamate receptor, metabotropic 7
Clock	clock circadian regulator
Trim9	tripartite motif-containing 9
Sdk2	sidekick cell adhesion molecule 2
Fbxo10	F-box protein 10
Gmnc	geminin coiled-coil domain containing
Numbl	numb-like
Abhd4	abhydrolase domain containing 4
Iqgap3	IQ motif containing GTPase activating protein 3
Per2	period circadian clock 2

bioRxiv preprint doi: https://doi.org/10.64898/2026.08.30.747939; this version posted September 1, 2026. The copyright holder for this preprint (which was not certified by peer review) is the author/funder, who has granted bioRxiv a license to display the preprint in perpetuity. It is made available under a CC-BY 4.0 International license.

Table S3. Description for quantitation and statistical tests analysis.

**Figure**	**Statistics method**	**Error bar**
**Fig. 1F**	P7 DKO vs. P7 TKO, n=3, ****P* = 0.0005, t = 10.57, df = 4; unpaired two-tailed Student's t-test	s.e.m.
**Fig. 1H**	**Aqp1**, P14 WT vs. P14 DKO, ns, *P* = 0.3086, t =1.838, df = 6; P14 WT vs. P14 TKO, **P* = 0.0214, t = 3.991, df = 6; P14 DKO vs. P14 TKO, ***P* = 0.0032, t = 5.884, df = 6.<br><br>**Atp1a2**, P14 WT vs. P14 DKO, ns, *P* = 0.8217, t =0.8322, df = 6; P14 WT vs. P14 TKO, **P* = 0.0472, t = 3.321, df = 6; P14 DKO vs. P14 TKO, **P* =0.0172, t = 4.186, df = 6.<br><br>**Nkcc1**, P14 WT vs. P14 DKO, ns, *P* = 0.1004, t =2.72, df = 6; P14 WT vs. P14 TKO, **P* = 0.0366, t = 3.53, df = 6; P14 DKO vs. P14 TKO, ***P* =0.0025, t = 6.172, df =6; n=3, Nested one-way ANOVA and Sidak multiple comparisons tests.<br><br>**Slc4a10**, P14 WT vs. P14 DKO, ns, *P* = 0.863, t =0.7448, df = 6; P14 WT vs. P14 TKO, **P* = 0.0187, t = 4.112, df = 6; P14 DKO vs. P14 TKO, **P* =0.0483, t = 3.302, df = 6.<br><br>**Ae2**, P14 WT vs. P14 DKO, ns, *P* = 0.412, t =1.593, df = 6; P14 WT vs. P14 TKO, ***P* = 0.0014, t = 6.883, df = 6; P14 DKO vs. P14 TKO, ****P* =0.0006, t = 8.126, df = 6.	s.e.m.
**Fig. 2G**	Basal body number, WT vs. DKO, ns, *P* = 0.9958, t = 0.2042, df = 48; WT vs. TKO, *****P* <0.0001, t = 21.47, df = 48; DKO vs. TKO, *****P* <0.0001, t = 19.44, df = 48; one-way ANOVA and Sidak multiple comparisons tests.	s.d.
**Fig. 3A**	**For WT,** DMSO vs. Shh, *****P* < 0.0001, t = 10.52, df = 8; WT DMSO vs. KAAD, ns, *P* = 0.0806, t = 2.687, df = 8; WT DMSO vs. Shh + KAAD, ns, *P* = 0.9981, t = 0.1618, df = 8.<br><br>**For DKO**, DMSO vs. Shh, **P* = 0.0182, t = 3.693, df = 8; DKO DMSO vs. KAAD, ns, *P* = 0.2766, t = 1.845, df = 8; DKO DMSO vs. Shh + KAAD, ns, *P* = 0.8200, t = 0.8211, df = 8.<br><br>**For TKO**, DMSO vs. Shh, ns, *P* = 0.1568, t = 2.242, df = 8; TKO DMSO vs. KAAD, ***P* = 0.0013, t = 5.744, df = 8; TKO DMSO vs. Shh + KAAD, ****P* = 0.0004, t = 6.727, df = 8; one-way ANOVA and Sidak multiple comparisons tests.	s.d.
**Fig. 3C**	P14 WT PBS vs. WT + Shh, ns, *P* = 0.11, t = 2.047, df = 4. P14 DKO PBS vs. DKO + Shh, ns, *P* = 0.302, t = 1.184, df = 4. P14	s.e.m.

bioRxiv preprint doi: https://doi.org/10.64898/2026.08.30.747939; this version posted September 1, 2026. The copyright holder for this preprint (which was not certified by peer review) is the author/funder, who has granted bioRxiv a license to display the preprint in perpetuity. It is made available under a CC-BY 4.0 International license.

	TKO PBS vs. TKO + Shh, ns, *P* = 0.1066, t = 2.075, df = 4; n =3, unpaired two-tailed Student's t-test.	
**Fig. 3D**	Ciliary Smo intensity, DKO WT PBS (n=54) vs. DKO Shh (n=61), ***P =0.0001, t = 3.961, df = 113; TKO WT PBS (n=39) vs. TKO Shh (n=65), ****P < 0.0001, t = 5.8, df = 102; unpaired two-tailed Student's t-test.	s.d.
**Fig. 3E**	For E14.5 *Gli1*, E14.5 WT vs. E14.5 DKO, ns, *P* = 0.9953, t = 0.2214, df = 6; E14.5 WT vs. E14.5 TKO, ns, *P* = 0.0161, t = 4.248, df = 6; E14.5 DKO vs. E14.5 TKO, *, *P* = 0.0127, t = 4.469, df = 6.<br><br>For P0 *Gli1*, P0 WT vs. P0 DKO, ns, *P* = 0.9905, t = 0.2808, df = 6; P0 WT vs. P0 TKO, ***P* = 0.0017, t = 6.616, df = 6; P0 DKO vs. P0 TKO, ** *P* = 0.0022, t = 6.335, df = 6.<br><br>For P14 *Gli1*, P14 WT vs. P14 DKO, ns, *P* = 0.9499, t = 0.5052, df = 6; P14 WT vs. P14 TKO, **P* = 0.0111, t = 4.593, df = 6; P14 DKO vs. P14 TKO, **P* = 0.0192, t = 4.088, df = 6; n=3, one-way ANOVA and Sidak multiple comparisons tests.	s.e.m.
**Fig. 3G**	Quantitation of apical Aqp1 expression in ciliated CPECs, DKO DMSO vs. TKO DMSO, ***P* = 0.0095, t = 4.154, df = 8; DKO DMSO vs. KAAD, **P* = 0.019, t = 4.154, df = 8; TKO DMSO vs. KAAD, ** *P* = 0.0099, t = 4.648, df = 8; DKO KAAD vs. TKO KAAD, ns, *P* = 0.1041, t = 2.959, df = 8; nested unpaired One-way ANOVA and Sidak multiple comparison.	s.d.
**Fig. 4B**	***Gmnc***, P0 WT vs. P0 DKO, ns, *P* = 0.9986, t = 0.148, df = 6; P0 WT vs. P0 TKO, * *P* = 0.0165, t = 4.224, df = 6; P0 DKO vs. P0 TKO, * *P* = 0.0141, t = 4.372, df = 6.<br><br>***Mcidas***, P0 WT vs. P0 DKO, ns, *P* = 0.7533, t = 0.9627, df = 6; P0 WT vs. P0 TKO, **** *P* < 0.0001, t = 26.3, df = 6; P0 DKO vs. P0 TKO, **** *P* < 0.0001, t = 25.33, df = 6.<br><br>***FoxJ1***, P0 WT vs. P0 DKO, ns, *P* = 0.9997, t = 0.08799, df = 6; P0 WT vs. P0 TKO, **** *P* < 0.0001, t = 11.13, df = 6; P0 DKO vs. P0 TKO, **** *P* < 0.0001, t = 11.04, df = 6; one-way ANOVA and Sidak multiple comparisons tests.<br><br>***Stil***, P0 WT vs. P0 DKO, ns, *P* = 0.9964, t = 0.2011, df = 6; P0 WT vs. P0 TKO, ** *P* = 0.0012, t = 7.078, df = 6; P0 DKO vs. P0 TKO, ** *P* = 0.0014, t = 6.877, df = 6.<br><br>***Plk4***, P0 WT vs. P0 DKO, ns, *P* = 0.9962, t = 0.2063, df = 6; P0 WT vs. P0 TKO, ** *P* = 0.0011, t = 7.186, df = 6; P0 DKO vs. P0 TKO, ** *P* = 0.0013, t = 6.979, df = 6.	s.e.m.

bioRxiv preprint doi: https://doi.org/10.64898/2026.08.30.747939; this version posted September 1, 2026. The copyright holder for this preprint (which was not certified by peer review) is the author/funder, who has granted bioRxiv a license to display the preprint in perpetuity. It is made available under a CC-BY 4.0 International license.

	Cep152, P0 WT vs. P0 DKO, ns, *P* = 0.9968, t = 0.07331, df = 6; P0 WT vs. P0 TKO, ** *P* = 0.0012, t = 7.093, df = 6; P0 DKO vs. P0 TKO, ** *P* = 0.0013, t = 7.019, df = 6.	
**Fig. 4F**	***Gmnc***, DKO+mock vs. TKO+mock, ** *P* = 5.651,t = 5.143, df = 6; TKO+mock vs. TKO+*miR34b/c*, *** *P* = 0.0004, t = 8.001, df = 6. ***Mcidas***, DKO+mock vs. TKO+mock, **** *P* < 0.0001, t = 25.51, df = 6; TKO+mock vs. TKO+*miR34b/c*, **** *P* < 0.0001, t = 21.99, df = 6. ***FoxJ1***, DKO+mock vs. TKO+mock, *** *P* = 0.0009, t = 6.854, df = 6; TKO+mock vs. TKO+*miR34b/c*, ** *P* = 0.0071, t = 34.636, df = 6; one-way ANOVA and Sidak multiple comparisons tests.	s.d.
**Fig. 4G**	WT *Gmnc*-3'UTR, *siGFP* vs. *miR34b* mimic, ns, *P* = 0.4358, t = 1.43, df = 8; *siGFP* vs. *miR34c* mimic, ** *P* = 0.0034, t = 4.929, df = 8; *siGFP* vs. *miR449a* mimic, *** *P* = 0.0005, t = 6.649, df = 8. For mutated *Gmnc*-3'UTR, *siGFP* vs. *miR34b* mimic, ns, *P* = 0.8671, t = 0.7241, df = 8; *siGFP* vs. *miR34c* mimic, ns, *P* = 0.797, t = 0.8649, df = 8; *siGFP* vs. *miR449a* mimic, ns, *P* = 0.9992, t = 0.1194, df = 8; one-way ANOVA and Sidak multiple comparisons tests.	s.e.m.
**Fig. 4H**	**Cent3 intensity**, DKO+*Scr shRN*A vs. TKO+*Scr shRN*A, ***P* = 0.0043, t = 5.143, df = 6; TKO+*Scr shRNA* vs. TKO+*Gmnc shRN*A, ***P* = 0.003, t = 5.505, df = 6; nested one-way ANOVA and Sidak multiple comparisons tests.	s.d
**Fig. 4I**	**Quantitation of FoxJ1 intensity**.<br>DKO+*Scr shRN*A vs. TKO+*Scr shRN*A, ** *P* = 0.0017, t = 6.644, df = 6; TKO+*Scr shRNA* vs. TKO+*Gmnc shRN*A, ***P* = 0.0087, t = 4.826, df = 6; nested one-way ANOVA and Sidak multiple comparisons tests.	s.d
**Sup Fig. S1B**	Brain weight quantification, n=3; DKO vs. TKO, **P* = 0.0253, t = 3.483, df = 4, unpaired two-tailed Student's t-test.	s.e.m.
**Sup Fig. S1D**	**Aqp1**, E14.5 WT vs. E14.5 DKO, ns, *P* = 0.1147, t =2.616, df = 6; E14.5 WT vs. E14.5 TKO, ns, *P* = 0.9919, t = 0.2660, df = 6; E14.5 DKO vs. E14.5 TKO, ns, *P* = 0.1532, t = 2.392, df = 6.<br>**Atp1a2**, E14.5 WT vs. E14.5 DKO, ns, *P* = 0.9919, t =0.2663, df = 6; E14.5 WT vs. E14.5 TKO, ns, *P* = 0.124, t = 2.556, df = 6; E14.5 DKO vs. E14.5 TKO, ns, *P* = 0.1634, t = 2.341, df = 6.<br>**Slc4a10,** E14.5 WT vs. E14.5 DKO, ns, *P* = 0.9904, t =0.2816, df = 6; E14.5 WT vs. E14.5 TKO, ns, *P* = 0.9986, t = 0.1452, df = 6; E14.5 DKO vs. E14.5 TKO, ns, *P* =0.9989, t = 0.1336, df = 6.	s.e.m.

bioRxiv preprint doi: https://doi.org/10.64898/2026.08.30.747939; this version posted September 1, 2026. The copyright holder for this preprint (which was not certified by peer review) is the author/funder, who has granted bioRxiv a license to display the preprint in perpetuity. It is made available under a CC-BY 4.0 International license.

	**Ae2**, E14.5 WT vs. E14.5 DKO, ns, *P* = 0.0382, t =3.494, df = 6; E14.5 WT vs. E14.5 TKO, ns, *P* = 0.7899, t = 0.8945, df = 6; E14.5 DKO vs. E14.5 TKO, ns, *P* = 0.1188, t = 2.589, df = 6.<br><br>**Nkcc1**, E14.5 WT vs. E14.5 DKO, ns, *P* = 0.2367, t =2.051, df = 6; E14.5 WT vs. E14.5 TKO, ns, *P* = 0.9004, t = 0.6556, df = 6; E14.5 DKO vs. E14.5 TKO, ns, *P* = 0.5262, t = 1.368, df = 6; n=3, Nested one-way ANOVA and Sidak multiple comparisons tests.	
**Sup Fig. S1E**	**Aqp1**, E14.5 WT vs. E14.5 DKO, ns, *P* = 0.9962, t =0.2057, df = 6; E14.5 WT vs. E14.5 TKO, **P* = 0.0127, t = 4.464, df = 6; E14.5 DKO vs. E14.5 TKO, **P* = 0.0103, t = 4.669, df = 6.<br><br>**Aqp1**, P14 WT vs. P14 DKO, ns, *P* = 0.3129, t =1.826, df = 6; P14 WT vs. P14 TKO, **P* = 0.0159, t = 4.259, df = 6; P14 DKO vs. P14 TKO, ***P* = 0.0027, t = 6.086, df = 6.<br><br>**Atp1a2**, E14.5 WT vs. E14.5 DKO, ns, *P* = 0.9898, t =0.2874, df = 6; E14.5 WT vs. E14.5 TKO, **P* = 0.0387, t = 3.484, df = 6; E14.5 DKO vs. E14.5 TKO, ns, *P* = 0.0550, t = 3.196, df = 6.<br><br>**Atp1a2**, P14 WT vs. P14 DKO, ns, *P* = 0.6348, t = 1.173, df = 6; P14 WT vs. P14 TKO, ***P* = 0.0096, t = 4.738, df = 6; P14 DKO vs. P14 TKO, **P* =0.0352, t = 3.565, df = 6.<br><br>**Nkcc1**, E14.5 WT vs. E14.5 DKO, ns, *P* = 0.6187, t = 1.201, df = 6; E14.5 WT vs. E14.5 TKO, **P* = 0.0127, t = 4.468, df = 6; E14.5 DKO vs. E14.5 TKO, ***P* = 0.0039, t = 5.67, df = 6;<br><br>**Nkcc1**, P14 WT vs. P14 DKO, ns, *P* = 0.4232, t = 1.57, df = 6; P14 WT vs. P14 TKO, *****P* < 0.0001, t = 12.19, df = 6; P14 DKO vs. P14 TKO, *****P* < 0.0001, t = 13.76, df =6;<br><br>**Ae2**, E14.5 WT vs. E14.5 DKO, ns, *P* = 0.9978, t = 0.1714, df = 6; E14.5 WT vs. E14.5 TKO, ns, *P* = 0.07, t = 3.003, df = 6; E14.5 DKO vs. E14.5 TKO, ns, *P* = 0.087, t = 2.832, df = 6;<br><br>**Ae2**, P14 WT vs. P14 DKO, ns, *P* = 0.0552, t = 3.193, df = 6; P14 WT vs. P14 TKO, **P* = 0.0107, t = 4.632, df = 6; P14 DKO vs. P14 TKO, ****P* =0.0007, t = 7.826, df = 6;<br><br>**Slc4a10,** E14.5 WT vs. E14.5 DKO, ns, *P* = 0.6672, t = 1.116, df = 6; E14.5 WT vs. E14.5 TKO, ***P* = 0.0029, t = 5.995, df = 6; E14.5 DKO vs. E14.5 TKO, ***P* = 0.0083, t = 4.879, df = 6;<br><br>**Slc4a10**, P14 WT vs. P14 DKO, ns, *P* = 0.9746, t = 0.3959, df = 6; P14 WT vs. P14 TKO, ***P* = 0.0028, t = 46.026, df = 6; P14 DKO vs. P14 TKO, ***P* = 0.002, t = 6.422, df = 6; n=3, Nested one-way ANOVA and Sidak multiple comparisons tests.	s.e.m.

bioRxiv preprint doi: https://doi.org/10.64898/2026.08.30.747939; this version posted September 1, 2026. The copyright holder for this preprint (which was not certified by peer review) is the author/funder, who has granted bioRxiv a license to display the preprint in perpetuity. It is made available under a CC-BY 4.0 International license.

**Sup Fig. 4B**	**Gmnc intensity**, E14.5 WT vs E14.5 DKO, ns, *P* = 0.294, t = 1.877, df = 6; E14.5 WT vs E14.5 TKO, *** *P* = 0.0006, t = 8.105, df = 6; E14.5 DKO vs E14.5 TKO, *** *P* = 0.0002, t = 9.983, df = 6; one-way ANOVA and Sidak multiple comparisons tests. one-way ANOVA and Sidak multiple comparisons tests.	s.e.m.
**Sup Fig. 4C**	**FoxJ1 intensity**, P0 WT vs. P0 DKO, ns, *P* =0.2298, t = 1.738, df = 280; P0 WT vs. P0 TKO, *****P* < 0.0001, t = 20.56, df = 280; P0 DKO vs. P0 TKO, *****P* < 0.0001, t = 23.08, df = 280; one-way ANOVA and Sidak multiple comparisons tests.	s.d.
**Sup Fig. 4D**	***Gmnc***, DKO+*Scr shRN*A vs. TKO+*Scr shRN*A, ***P* = 0.0043, t = 5.143, df = 6; TKO+*Scr shRN*A vs. TKO+*Gmnc shRN*A, ***P* = 0.003, t = 5.505, df = 6.<br><br>***Mcidas***, DKO+*Scr shRN*A vs. TKO+*Scr shRN*A, *****P* < 0.0001, t = 12, df = 6; TKO+*Scr shRN*A vs. TKO+*Gmnc shRN*A, ****P* = 0.0007, t = 7.302, df = 6.<br><br>***FoxJ1***, DKO+*Scr shRN*A vs. TKO+*Scr shRN*A, ***P* = 0.0038, t = 5.256, df = 6; TKO+*Scr shRN*A vs. TKO+*Gmnc shRN*A, **P* = 0.0326 t = 3.3, df = 6; one-way ANOVA and Sidak multiple comparisons tests.	s.d

bioRxiv preprint doi: https://doi.org/10.64898/2026.08.30.747939; this version posted September 1, 2026. The copyright holder for this preprint (which was not certified by peer review) is the author/funder, who has granted bioRxiv a license to display the preprint in perpetuity. It is made available under a CC-BY 4.0 International license.

Table S4. Primer sequences for mouse genotype, qRT-PCR and miRNA mimics

Table S4.1 Primer sequences for mouse genotype

Mouse strain	Name	Sequence	Band size
miR-34/449 *TKO*	*mir-34b/34c*-WT-F	GCCTCCTGTGAATCGTCATT	mir-34b/34c WT: 264bp
	mir-34b/34c-Common-R	GAGATTTTCGTGGCGCTTTA	mir-34b/34c null: 155bp
	mir-34b/34c-KO-F	GCGGCCGCATAACTTCGTAT	

bioRxiv preprint doi: https://doi.org/10.64898/2026.08.30.747939; this version posted September 1, 2026. The copyright holder for this preprint (which was not certified by peer review) is the author/funder, who has granted bioRxiv a license to display the preprint in perpetuity. It is made available under a CC-BY 4.0 International license.

Table S4. Primer sequences for mouse genotype, qRT-PCR and miRNA mimics
Table S4.2 Primer sequences for real time PCR analyses

Gene	Primer	Primer sequence
β-Actin	β-Actin-F	GATCTGGCACCACACCTTCT
	β-Actin-R	GGGGTGTTGAAGGTCTCAAA
Gli1	Gli-1-F	TACCATGAGCCCTTCTTTAGGA
	Gli-1-R	GCATCATTGAACCCCGAGTAG
Gli2	Gli-2 -F	TAC CTC AAC CCT GTG GAT GC
	Gli-2 -R	CTA CCA GCG AGT TGG GAG AG
Ptc1	Ptch1-F	ATC TCG AGA CCA ACG TGG AG
	Ptch1-R	TAG CGC CTT CTT CTT TTG GA
Hhip	Hhip-F	TGAAGATGCTCTCGTTTAAGCTG
	Hhip-R	CCACCACACAGGATCTCTCC
Shh	Shh-F	AAAGCTGACCCCTTTAGCCTA
	Shh-R	TTCGGAGTTTCTTGTGATCTTCC
Stil	Stil-F	CCTTGTGAGAGTAGGACGCC
	Stil-R	TGGGAGGATCTCTTCACTGGA
Plk4	Plk4-F	GCTTCTGAGAGAGTGTGCGTCT
	Plk4-R	GTCTGTCAGCAAGAGGAAAGCC
Cep152	Cep152-F	CTACACAGCAGGCTGAGAAGGA
	Cep152-R	TGAGGTGGTCTTTCTCCAAGCC
Gmnc	Gmnc-F	TCTGGAAGAGAAGGCCAAGA
	Gmnc-R	CCCAGGTTGTTCCTCACAGT
Mcidas	Mcidas-F	GTGGAAGTCCTTTTCGGGATGC
	Mcidas-R	TAGGAGACGCTTCGGTTCCGAG
FoxJ1	Foxj1-F	AAGGTAACTTTGACTGGGAG
	Foxj1-R	GGACTGTAAGATCCACATCTC
Aqp1	Aqp1-F	CTTGCCATTGGCTTGTCTGTGG
	Aqp1-R	CCAGTGGTTTGAGAAGTTGCGG
Atp1a2	Atp1a2-F	ACAGGAACCCTAAGGTGGCAGA
	Atp1a2-R	CGCCTTTCATCACCAGCACATG
Slc4a2	Slc4a2-F	CAGCAGTGCTAACATGGACTACC
	Slc4a2-R	AGAAGGCGGTAGCACAACACTG
Slc4a10	Slc4a10-F	ACACAGCGGACCTGAACTCCAG
	Slc4a10-R	CAGGAACGATGCTAAGCACTGC
Slc12a2	Slc12a2-F	ATCCGCCTGAAGGAAGGACT
	Slc12a2-R	TGCCATCCTCTTCCTCATCTTTC
Trpm3	Trpm3-F	TCTACAACGCACCCATCGTGAAG
	Trpm3-R	GCGCTCCATCTTCACTAACACG

bioRxiv preprint doi: https://doi.org/10.64898/2026.08.30.747939; this version posted September 1, 2026. The copyright holder for this preprint (which was not certified by peer review) is the author/funder, who has granted bioRxiv a license to display the preprint in perpetuity. It is made available under a CC-BY 4.0 International license.

Table S4. Primer sequences for mouse genotype, qRT-PCR and miRNA mimics

Table S4.3 sequences for miRNA mimics

miRNA name	mimic name	Sequence
miR-34b	miR-34b 5p mimic	AGGCAGUGUAAUUAGCUGAUUGU
	miR-34b 3p mimic	AAUCACUAACUCCACUUCCUUC
miR-34c	miR-34c 5p mimic	AGGCAGUGUAGUUAGCUGAUUGC
	miR-34c 3p mimic	AAUCACUAACCACACAGCUUGG
miR-449a	miR-449a 5p mimic	UGGCAGUGUAUUGUUAGCUGGU
	miR-449a 3p mimic	CAGCUAACAUGCGACUGUUAUC
